# Syndemic and Synergistic Effects of Intimate Partner Violence, Crystal Methamphetamine, and Depression on HIV Sexual Risk Behaviors among Women Who Inject Drugs in Indonesia

**DOI:** 10.1007/s11524-019-00352-6

**Published:** 2019-03-14

**Authors:** Claudia Stoicescu, Rima Ameilia, Irwanto, Ignatius Praptoraharjo, Mietta Mahanani

**Affiliations:** 1 Centre for Evidence-Based Intervention, Department of Social Policy and Intervention, University of Oxford, Barnett House, 32 Wellington Square, Oxford, OX1 2ER UK; 2 Department of Criminology, University of Indonesia, Depok, Indonesia; 3 HIV and AIDS Research Centre, Atma Jaya Catholic University, Jakarta, Indonesia; 4 Center for Health Policy and Management, Faculty of Medicine, Gadjah Mada University, Yogyakarta, Indonesia; 5 Faculty of Public Health, University of Indonesia, Depok, Indonesia

**Keywords:** Syndemics, Synergism, Women who inject drugs, HIV, Sexual risk behavior

## Abstract

Women who inject drugs are disproportionately affected by co-occurring intimate partner violence (IPV), poor mental health, and substance use. Less is known about the potentially synergistic effects of these factors on women’s HIV risk behavior, and no known studies in Asia examine these relationships. This study assessed the additive and interactive effects of exposure to syndemic IPV, depressive symptoms and non-injection crystal methamphetamine (crystal meth) on HIV sexual risk behaviors in the largest cross-sectional sample of women who inject drugs in Indonesia. Seven hundred thirty-one women aged ≥ 18 years, injecting drugs in the preceding 12 months, and residing in Greater Jakarta or Bandung, West Java, were recruited using respondent-driven sampling (RDS). Twenty-six percent of women experienced concurrent IPV, crystal meth use and depressive symptoms. In multivariate logistic regressions controlling for sociodemographic confounders, all three factors were significantly positively associated with sexual risk outcomes. In adjusted marginal effects models, concurrent experience of IPV, crystal meth use and depressive symptoms was associated with increases in the prevalence of HIV risk outcomes: STI symptomatology (from 12% to 60%), inconsistent condom use (from 3% to 22%), and engagement in survival sex work (from 6% to 25%). Statistically significant interaction was detected on both multiplicative and additive scales. Specifically, an interaction was observed on the multiplicative scale between depressive symptoms and crystal meth on STI symptomatology (OR = 2.61; 95% CI = 1.24, 5.48; *p* = 0.011). There was also evidence of additive interaction, with most observed joint effects being greater than additive. Specifically, significant positive interaction was observed between IPV and crystal meth on inconsistent condom use (AP = 0.38, *p* < 0.05); depressive symptoms and crystal meth on STI symptomatology (RERI = 2.04, *p* < 0.001; AP = 0.61, *p* < 0.001) and survival sex (RERI = 1.20, *p* < 0.01; AP = 0.53, *p* < 0.01); and IPV and depressive symptoms on STI symptomatology (RERI = 3.01, *p* < 0.01; AP = 0.52, *p* < 0.001; *S* = 2.70, *p* < 0.01) and survival sex (RERI = 1.21, *p* < 0.05; AP = 0.40, *p* < 0.05). This study provides new empirical evidence showing that the syndemic conditions of IPV, depressive symptoms and crystal meth consumption interact synergistically to increase women’s HIV risk. Interventions that consider the full scope of syndemic vulnerabilities, rather than addressing individual conditions separately, may be essential.

## Introduction

Women account for half of the 37 million people estimated to be living with HIV globally [1]. In regions where HIV is concentrated among key at-risk populations, the proportion of newly diagnosed women is increasing [2]. People who inject drugs are one such at-risk population, with women constituting an estimated one-third of all drug injectors [3].

Extensive research, largely conducted in the USA, has focused on the clustering of substance use, violence, and HIV and AIDS, described together as the “SAVA syndemic” [4], as the key locus of intersecting risk for women who inject drugs [5–7]. A syndemic refers to two or more overlapping comorbidities, sustained by structural contexts of inequality, structural violence, and stigmatization that accumulate and interact synergistically to create excess burden of health adversity in a population [9]. Compared to their male counterparts and vis-à-vis women in the general population, women who inject drugs are disproportionately affected by all three conditions, often concurrently, which facilitates added HIV vulnerability [5, 8]. 

The mechanisms that link substance use, violence, and HIV among marginalized women are both direct and indirect. For instance, meta-analytic evidence has shown that the influence of IPV and substance use among women is complex and bi-directional [10]. Risky drug-using behaviors may be triggered by the traumatic experience of IPV or the mental health consequences of victimization, including depression and post-traumatic stress disorder [5, 11–13]. Substance use, in turn, is associated with elevated mental health problems [14, 15]. Psychiatric comorbidities, particularly depression, play a key role in perpetuating and mediating overlapping HIV vulnerabilities because they often co-occur and have mutually reinforcing relationships with both substance use and IPV [14, 16]. Women who inject drugs are also at higher risk of experiencing IPV and acquiring HIV [17, 18]. Specifically, sexual violence has been associated with HIV and other sexually transmitted infections (STIs) among drug-using women via biological mechanisms such as vaginal and anal lacerations resulting from aggressive sex [19, 20]. Indirectly, IPV increases the risk of condomless and high-risk sex by normalizing an unequal dynamic of control and fear that thwarts women’s capacity to negotiate condom use [21, 22].

Drawing on the original conceptualization of syndemic theory in 1996 [9, 23], since further articulated by Tsai and others [24–26], two key aspects of the approach must be considered in empirical investigations: (1) the co-occurrence of conditions in a given population (disease concentration) and (2) the interaction of conditions to produce mutually detrimental effects on health at the population and individual levels (disease interaction). Despite the proliferation of studies examining the *concentration* of risk factors, the evidence base for testing whether such factors *interact* synergistically to amplify health adversity among women who inject drugs remains limited. For instance, among 71 quantitative studies investigating the empirical merit of the syndemic framework identified by a recent systematic review, 57 studies (80%) tested the disease concentration concept using some variation of a sum score corresponding to the total count of psychosocial problems [24, 25]. None of the studies went beyond the count variable approach to explore interaction between comorbidities. Crucially, of the 10 (14%) studies that focused on female populations, including women who use and/or inject drugs, none tested for the interaction of concurrent health problems on women’s HIV outcomes.

In the absence of experimental and longitudinal studies that test the syndemic and synergistic effects of co-occurring risk factors on outcomes of interest, Tsai and others argue that analyses of cross-sectional data employing robust econometric methods can provide compelling evidence for probable synergistic risk factors that merit further investigation [24]. To this end, epidemiologists have argued for an expansion of the assessment of interactions based purely on product terms in multiplicative models of cross-sectional data to also include additive interaction [24, 27, 28]. Briefly, statistical interaction is said to be present on the multiplicative scale when the joint effect of risk factors differs from the product of the effects of the individual factors [29]. Statistical interaction can also be detected on the additive scale, and is present when the joint effect of risk factors differs from the sum of the effects of the individual factors. Additive interaction, also known as synergism, refers to the interdependent action of two or more risk factors to produce an effect [30]. It has been argued that the latter concept is an appropriate way to statistically approximate the underlying causal mechanisms on a given outcome in studies of health behavior, and thus has more direct public health relevance [29, 31]. Moreover, existing research on the application of a syndemic framework to understand HIV risk dynamics among women who inject drugs is limited by its geographic scope, with most studies conducted in the USA, and to a lesser extent, sub-Saharan Africa [32]. To achieve optimal public health impact, it is necessary to broaden the geographical scope of studies assessing syndemics among women who use and inject drugs to include resource-limited geographical settings, particularly regions with injection-driven HIV epidemics, such as Asia and Eastern Europe.

### The National Context in Indonesia

Asia has a critical gap in research on drug-using women. The region is home to the largest absolute numbers of women who inject drugs (1.5 million) and amphetamine-dependent women (2.4 million) globally [2]. A sprawling archipelago comprising over 17,000 islands, Indonesia is home to the world's fourth largest population and a key destination and transshipment point for drug trafficking to and from other parts of South East Asia, Africa, and the Middle East [33]. In the past decade, Indonesia has also emerged as a manufacturing hub for amphetamine-type stimulants [34]. The country is facing an expanding HIV epidemic concentrated among key affected populations, including people who use and inject drugs, sex workers and their clients, transgender persons, and men who have sex with men [1].

Recent bio-behavioral surveillance in Indonesia identified people who inject drugs as the only key population among whom HIV sexual risk behaviors had escalated since 2013 [35]. Heterosexual transmission has played a growing role in Indonesia’s HIV epidemic, with women comprising an increasing proportion of incident HIV cases [36].

Previous literature addressing women who inject drugs in the Indonesian context, whilst not focused on HIV risk only, provides helpful indications of potential risk factors particular to this group that merit further attention. Specifically, health and psychosocial disparities in IPV and mental health experienced by Indonesian women who inject drugs in conjunction with elevated rates of HIV provide an indication of where one may begin investigating processes driving HIV risk in this population [38–42]. In an earlier analysis, we detected 41.6% self-reported HIV prevalence in a sample of women who inject drugs in Indonesia in 2015 [37], which is approximately 13% higher than HIV prevalence (28.8%) among a mostly male national sample of people who inject drugs from the same year [35]. We also found that IPV prevalence among women who inject drugs was up to 24 times higher than prevalence among women in the Indonesian general population [42]. Crucially, women’s experience of IPV victimization independently predicted elevated sexual risk behavior.

Depression is another common health disparity that disproportionately affects people who inject drugs in Indonesia. The prevalence of severe depressive symptoms among a sample of people who inject drugs recruited from Jakarta and Denpasar was nearly five times higher (33%) than prevalence found in a general Indonesian population sample (7%) [43, 44]. Depression has been associated with recent substance use among Indonesian people who use drugs and have HIV [44], but is understudied among women. In international research, depression has been shown to play a role in increasing women’s substance use and in inhibiting their ability to discern and navigate risky situations [16].

The potential influence of non-injecting drugs, particularly methamphetamine and amphetamine-type stimulants, which are causally linked with HIV sexual risk and HIV infection [45, 46], must also considered within a syndemic model for women who use drugs in the Indonesian context. Recent biological and behavioral surveillance in six major cities suggested that use of non-injecting drugs, particularly stimulants, may be indirectly contributing to the country’s HIV epidemic [38, 47]. Amphetamine-type stimulants, including crystal meth, are the most widely used substances in Indonesia, consumed by more than 1.3 million people, 294,000 (23%) of whom are women [48]. Research has shown that drug use categories are rarely mutually exclusive [49], with “bridging” between injecting and non-injecting populations often facilitating HIV transmission via overlapping social and sexual networks [49]. This appears to hold true in the Indonesian context, where in 2017 HIV prevalence among users of crystal meth with no prior injecting experience was 3.4%, but surged to 35% among those who used crystal meth and injected drugs [47].

A better understanding of the potential cumulative and interactive effects of key risk factors for HIV sexual transmission risk among women who inject drugs is essential for elucidating pathways to HIV and developing effective tailored interventions for this population. This study aims to address this research gap. We conducted Indonesia’s largest study of women who inject drugs to assess (1) whether depressive symptoms, IPV, and crystal meth use are independently associated with elevated HIV sexual risk behavior; (2) the potential additive effects of aggregate exposure to one or more of these conditions on women’s HIV sexual risk behaviors; and (3) the extent to which these conditions may interact on the multiplicative and/or additive scales to increase women’s odds of participation in HIV sexual risk behaviors beyond each condition’s individual effects.

## Methods

### Study Design and Sampling

The study methods have been described in detail elsewhere [42]. Briefly, between September 2014 and June 2015, 731 women were recruited using respondent-driven sampling (RDS) [50] from Greater Jakarta, which comprises Indonesia’s capital, Jakarta and its metropolitan areas Bogor, Tangerang, Depok and Bekasi, and Bandung, the provincial capital of West Java. Eligibility criteria included the following: being female; ≥ 18 years of age; injecting illicit drugs in the preceding 12 months; residing in one of the study areas; having a valid recruitment referral; and providing voluntary informed consent. A diverse group of 20 initial recruits (“seeds”), diverse in relation to age, socioeconomic status, levels of risk behavior, and known HIV status, were selected and asked to enlist up to three eligible peers in the study. Chain-like waves of recruitment continued until the desired sample size was reached. Participants received a primary incentive of 75,000 Indonesian Rupiah (~ 5 USD) for participating in the interview and a secondary incentive of 25,000 Indonesian Rupiah (~ 2 USD) per eligible peer recruited. Women completed an interviewer-administered survey eliciting information on sociodemographic and behavioral characteristics using tablets equipped with Open Data Kit, an open-source application for mobile data collection and management [51]. Seven female peer fieldworkers were trained in questionnaire administration using mobile-assisted technology, ethics, and health and safety. Interviews lasting approximately 1 h were conducted at locations deemed safe by participants, such as offices of non-governmental organizations or participants’ homes.

The study was anonymous; all participants were encouraged to use a pseudonym. Participants provided verbal and written voluntary informed consent. Consent forms used plain language and included clear explanations of the nature and purpose of the research, limits to confidentiality, and explicit statements regarding participants’ rights to opt-out at any time. Interviewers read and discussed consent forms verbally to ensure that participants understood the information needed to provide informed consent, regardless of literacy level. Strict confidentiality was maintained, except where women requested assistance or service referrals. In the case that information disclosed suggested that a participant was at risk of significant harm, interviewers discussed concerns with the participant and offered service referrals. The Institutional Review Boards of Oxford University (ref no: SSD/CUREC2/13-23) and Atma Jaya University (ref no: 1114/III/LPPM-PM.10.05/11/2013) approved the study protocol.

### Measurement

#### HIV Risk Behavior Outcomes

The primary outcome of interest was HIV sexual risk behavior. Sexual risk behavior was defined using three behaviors associated with HIV infection among women who inject drugs and analyzed separately for each construct: (1) inconsistent condom use; (2) sexually transmitted infection (STI) symptomatology; and (3) survival sex work.

*Inconsistent condom use* was based on reporting less than 100% condom use for vaginal and/or anal sex in the previous 12 months with steady, casual, and transactional partners. Consistent condom users were defined as those who reported using condoms “every time,” and inconsistent users as those reporting using condoms “most of the time,” “sometimes,” or “never.”

*STI symptomatology* was measured using participant self-report of easily recognized symptoms, based on World Health Organization guidelines [52]. Sexually active participants reporting ≥ 2 of 6 current symptoms (e.g., “sores, blisters, and/or ulcers on or in the vagina,” “unusual vaginal discharge, such as pus or a thick and/or sticky liquid from the genital area”) were coded as 1 = “having STI symptoms.”

*Survival sex work* was assessed by asking participants whether they traded sex for any reason in the previous year. Those who responded in the affirmative were further asked whether they traded sex for one or more of the reasons listed. The multiple choice of items included “money,” “drugs,” “luxury items” (e.g., phones or jewelry), “basic needs” (shelter, food, or other subsistence needs), “other (specify).” Based on the latter question, survival sex work was the exchange of sex for drugs, shelter, or other commodities as a means of basic subsistence in the previous 12 months.

#### Syndemic Factors

*Non-injection crystal meth use* was measured by asking participants which illicit and/or illegal substances they used at least once in the previous 12 months. A dichotomous variable was created to reflect any past-year use of non-injection crystal meth in addition to women’s injecting drug use.

*Intimate partner violence* was assessed using the Revised Conflict Tactics Scale (CTS2) short form [53, 54]. Reliability of the CTS2 for this sample was *α* = 0.85. CTS2 items ask respondents to indicate how often each event took place within a referent period [55]. Participants were asked about violence perpetrated by a current or former intimate partner, including partners within formal and informal partnerships, in the preceding 12 months. Responses to victimization items on the instrument’s psychological, physical, injurious, and sexual subscales were summed, combined, and dichotomized into any vs no IPV victimization.

*Depressive symptoms* were measured using the Revised Center for Epidemiologic Studies Depression (CESD-R) scale [56]. The CESD-R is validated and used widely across settings, including among samples of people who inject drugs in Indonesia [44, 57]. The scale measures symptoms of depression in the previous 2 weeks, as defined by the American Psychiatric Association Diagnostic and Statistical Manual, fifth edition. Possible scores for the 20-item scale range from 0 to 60, with a cut-off score of 16 or above indicating the presence of clinically significant depressive symptoms. Responses to the 20 questions were summed, combined, and dichotomized as either below or above 16. For this sample, the reliability of the CESD-R was *α* = 0.90.

#### Sociodemographic Characteristics

The following socioeconomic and background information was collected: *age*, *relationship status*, *employment status*, *education level*, *housing status*, *individual monthly income*, *and whether they had any dependent children* in the household or other dependents for whom they were responsible, and *known HIV status*. Individual monthly income was classified as being either below or above the mean national income in Indonesia [58]. Housing status was assessed by asking women about their current living arrangements. Women were coded as “homeless/unstably housed” if they lived on the street, in public spaces (i.e., train station) or in temporary or transitional accommodation, and “stably housed” if they lived in a family home, rental house/apartment, or long-term single-room rental accommodation (*kos-kosan*).

### Statistical Analysis

Data were analyzed in Stata 14.2 (StataCorp, College Station, TX) in four stages:

1. First, population proportions and 95% confidence intervals were computed for study outcomes and syndemic variables using RDS-II generated weights in the RDS analysis software for Stata [59, 60], and reported separately for each survey city. Since the majority of participants in our study resided in either one of the two cities, forming two network samples with minimal across-group recruitment, we followed the standard RDS practice of reporting estimates for each network sample individually, rather than combining them into an overall estimate [61]. However, in order to retain the power and precision of the original calculated sample size, bivariate and multivariate analyses were performed on the complete, unweighted sample combining data from the two survey cities. Unweighted frequencies for all variables were also calculated on the merged dataset.
2. Second, bivariate and multivariate logistic regressions were performed to explore associations between syndemic variables, potential sociodemographic confounders, and each of the three HIV risk behavior outcomes. Variables that attained a significant level < 10% in bivariate analyses were retained in subsequent multivariate analyses [62].
3. Third, three sets of marginal effects models (one for each outcome) were conducted to test the additive effects of cumulative syndemic exposures on sexual risk behaviors, with significant covariates from stage 2 held at mean values.
4. Fourth, to test the assertion that concurrent health and social problems interact to increase HIV risk behavior, measures for both multiplicative and additive interaction were computed. Multiplicative interaction reflects the degree to which odds of an exposure are *multiplied* in individuals with a given risk factor compared to those without it. To assess interaction on the multiplicative scale, we tested two- and three-way product terms of syndemic risk factor exposures on each of the three behavioral outcomes. Additive interaction refers to the interdependent action of two or more factors to produce or prevent an effect, and assesses whether the combined effect of two exposures is more or less than the sum of their separate effects [29, 63]. In epidemiological studies, it is measured using the difference of risk differences, also known as the interaction contrast (IC) [64]. Three indices developed by Rothman [65] were computed to test interaction contrast using the *IC* statistical package for Stata [66]. This statistical package implements the procedure described in Hosmer and Lemeshow (1992) and Andersson et al. (2005) for calculating metrics of additive interaction, 95% confidence intervals, and statistical significance [67, 68]. To enable the measurement of synergism metrics, three dummy variables were created to calculate the joint effects of each pair of syndemic conditions [29, 68]. These were the following: (1) IPV and depression, (2) IPV and crystal meth use, and (3) crystal meth use and depression. Dummy variables were coded as follows: “0” = presence of neither condition (reference category), “1” = presence of condition A, but not condition B, “2” = presence of condition B, but not condition A, and “3” = presence of both conditions. A series of logistic regressions were run with the sexual risk outcomes regressed separately on each of the 3 dummy coded variables, adjusting for significant confounders (9 regression models in total).

To quantify the amount of interaction on the additive scale, three indices were calculated based on the OR estimates [29, 69]:

(i) RERI, the relative excess risk due to interaction in relation to the level of risk at no exposure, is calculated as the difference between the expected risk and the observed risk:

$$ \mathrm{RERI}={\mathrm{OR}}_{++}-{\mathrm{OR}}_{+-}-{\mathrm{OR}}_{-+}+1. $$

(ii) AP, which computes the attributable proportion of risk due to interaction among those with both exposures:

$$ \mathrm{AP}=\mathrm{RERI}/{\mathrm{OR}}_{++}. $$

(iii) *S*, the synergy index, which measures the interaction between two risk factors expressed as the ratio of the relative excess risk for the combined effect of the risk factors and the sum of the relative excess risks for each separate effect of the two risk factors:

$$ S=\left[{\mathrm{OR}}_{++}-1\right]/\left[\right({\mathrm{OR}}_{+-}-1\left]+\left({\mathrm{OR}}_{-+}-1\right)\right]. $$

As recommended by the Strengthening the Reporting of Observational Studies in Epidemiology (STROBE) Statement, the separate effect of each exposure was reported in addition to the joint effect compared with the unexposed group as a reference category [70]. We were unable to test for additive interaction beyond two exposures, as there is no consensus in the literature on a rigorous way to conduct this computation [71]. Some epidemiologists consider any departure from 0 (in the case of RERI and AP) or 1 (in the case of *S*) as evidence for the presence of interaction. However, there are now several methods to derive CIs and *p* values around these measures [67, 68, 72] and software that easily calculates these additional computations. Therefore, for this study, statistically significant RERI > 0, AP > 0, or *S* > 1 indicated the presence of interaction on the additive scale.

## Results

### Sociodemographic Characteristics

Across the two study cities, the majority of participants were under 35 years of age (64.7%), and 12.6% were 24 years or younger. 62.7% were married or in a steady relationship, 13.9% were single (never married), 13.5% were divorced, and 9.9% were widowed. The majority of women (56.5%) had children or other dependents for whom they were responsible. 79.8% of women completed at least a high school education, and 44.3% were unemployed. Self-reported, unweighted HIV prevalence among women in the combined sample was 46.7% (Table 1).

**Table 1:** Sample characteristics, syndemic variables, and sexual risk practices among women who inject drugs in Indonesia, unadjusted estimates

Independent variables	Total *N* = 731	
	*N*	%
Age groups		
≤ 24 years	92	12.6
25–34 years	473	64.7
≥ 35 years	166	22.7
Education level (highest attained)		
Less than high school	148	20.2
High school or higher	583	79.8
Employment status		
Currently working	407	55.7
Not currently working	324	44.3
Individual monthly income (million IDR)		
≤ 3.8 (approx. 285 USD)	398	54.5
> 3.8 (approx. 285 USD)	333	45.5
Relationship status		
Married/steady relationship	458	62.7
Single, never married	102	13.9
Divorced	99	13.5
Widowed	72	9.9
Dependent children or other dependents		
Yes	413	56.5
No	318	43.5
Housing status		
Homeless/unstable housing	39	5.3
Stable housing	692	94.7
Self-reported HIV status		
Positive	341	46.7
Negative/unknown	390	53.3
Intimate partner violence		
Any IPV in the past year	492	67.3
No IPV in the past year	239	32.7
Crystal methamphetamine use		
Yes	491	67.2
No	240	32.8
Depressive symptoms (CESD-R ≥ 16)		
Yes	478	65.4
No	253	34.6
STI symptomatology		
Yes	324	44.3
No	407	55.7
Condom use consistency		
Consistent	135	18.5
Inconsistent	596	81.5
Survival sex work		
Yes	174	23.8
No	557	76.2

*IDR*, Indonesian Rupiah; *USD*, US dollars; *STI*, sexually transmitted infection; *CESD-R*, Revised Center for Epidemiologic Studies Depression scale

### Sexual Risk Behavior and Syndemic Factors

Table 2 presents RDS-weighted estimations and 95% confidence intervals (CI) for sexual risk outcomes and syndemic variables, i.e., IPV, depressive symptoms, and crystal meth use, by city. There were notable city differences in relation to two of the three sexual risk outcomes assessed. First, the prevalence of inconsistent condom use was more than 10% higher in Greater Jakarta (68.8%; 95% CI = 64.8, 72.5) relative to Bandung (58.2%; 95% CI = 50.3, 65.6). Second, STI symptomatology was reported by more than half of the women recruited from Greater Jakarta (52.8%; 95% CI = 48.6, 56.8), and 1 in 5 of the women recruited from Bandung (21.2%; 95% CI = 15.7, 28.1). The prevalence of survival sex work was 18.1% (95% CI = 12.8, 25.0) in Bandung and 16.8% (95% CI = 14.3, 19.6) in Jakarta.

**Table 2:** RDS-weighted estimations and 95% confidence intervals for sexual risk outcomes, depressive symptoms, IPV, and substance use among women who inject drugs in Indonesia

	Greater Jakarta (*n* = 572)				Bandung (*n* = 159)			
	*N*	Unweighted %	RDS-weighted %	95% CI	*N*	Unweighted %	RDS-weighted %	95% CI
Intimate partner violence								
Any IPV in the past year	401	70.1	68.9	65.0, 72.6	91	57.2	55.9	48.0, 63.5
No IPV in the past year	171	29.9	31.1	27.3, 35.0	68	42.8	44.1	36.5, 52.0
Non-injection crystal methamphetamine use								
Yes	417	72.9	69.1	65.0, 72.9	74	46.5	50.9	41.3, 56.9
No	155	27.1	30.9	27.1, 35.0	85	53.5	49.1	43.0, 58.6
Depressive symptoms (CESD-R ≥ 16)								
Yes	388	67.8	67.0	63.0, 70.7	90	56.6	55.6	47.7, 63.3
No	184	32.2	33.0	29.2, 37.0	69	43.4	44.4	36.7, 52.2
STI symptomatology (≥ 2)								
Yes	287	50.2	52.8	48.6, 56.8	37	23.3	21.2	15.7, 28.1
No	285	49.8	47.2	43.2, 51.3	122	76.7	78.8	71.9, 84.3
Condom use consistency (past 12 months)								
Consistent	169	29.5	31.2	27.5, 35.2	68	42.8	41.8	34.4, 49.7
Inconsistent	403	70.5	68.8	64.8, 72.5	91	57.2	58.2	50.3, 65.6
Survival sex work (past 12 months)								
Yes	146	25.5	16.8	14.3, 19.6	28	17.6	18.1	12.8, 25.0
No	426	74.5	83.2	80.3, 85.6	131	82.4	81.9	75.0, 87.2

*CI*, confidence intervals; *IDR*, Indonesian Rupiah; *USD*, US dollars; *STI*, sexually transmitted infection; *CESD-R*, Revised Center for Epidemiologic Studies Depression scale

City differences were also observed for the three syndemic factors assessed (i.e., IPV, depressive symptoms, and crystal meth use). The prevalence of depressive symptoms was higher in Greater Jakarta (67.0%; 95% CI = 63.0, 70.7) than Bandung (55.6%; 95% CI = 47.7, 63.3). Furthermore, prevalence figures of past-year IPV victimization among women in Greater Jakarta (68.9%; 95% CI = 65.0, 72.6) were over 10% higher than those reported by women in Bandung (55.9%; 95% CI = 48.0, 63.5). Non-injection crystal meth was used by 69.1% (95% CI = 65.0, 72.9) of women in Greater Jakarta and 50.9% (95% CI = 41.3, 56.9) of women in Bandung.

**Fig. 1:**
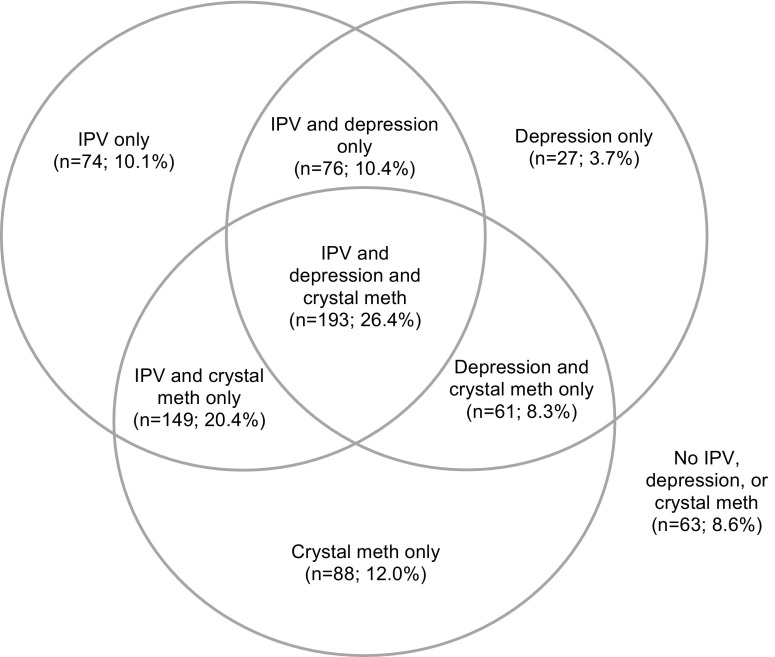
Overlap in exposures to syndemic conditions (past-year intimate partner violence, depressive symptoms, non-injection crystal meth use) among women who inject drugs, unweighted estimates

The overlap in exposures to syndemic problems in the aggregated, unweighted sample is illustrated in Fig. 1. Between 3.7 and 12.0% of women reported one of either depression, IPV, or crystal meth use. 8.3–20.4% of women experienced two simultaneously co-existing conditions. Notably, more than one quarter of women in the sample (26.4%) were exposed to all three conditions concurrently.

### Associations between Syndemic Factors and HIV Risk Behaviors

Results from bivariate analyses between variables listed in Table 1 and HIV sexual risk outcomes are displayed in Table 3. The following covariates were significantly associated with all three HIV risk behavior outcomes at *p* < 0.1 and were thus retained in multivariate models: self-reported HIV-positive status, homelessness/unstable housing, lower than high school educational attainment, relationship status, higher individual monthly income, and having children and/or other dependents.

**Table 3:** Bivariate associations between syndemic factors, sociodemographic characteristics, and HIV sexual risk behaviors among women who inject drugs in Indonesia

Independent variables (categories)	STI symptomatology			Inconsistent condom use			Survival sex work		
	ORs	95% CIs	*p* value	ORs	95% CIs	*p* value	ORs	95% CIs	*p* value
Past-year intimate partner violence									
Yes	3.16	2.25, 4.43	< 0.001	3.83	2.27, 6.47	< 0.001	1.86	1.25, 2.76	0.002
No	1			1			1		
Depressive symptoms									
Yes (CESD-R ≥ 16)	3.05	2.19, 2.24	< 0.001	2.44	1.55, 3.85	< 0.001	2.34	1.57, 3.51	< 0.001
No (CESD-R < 16)	1			1			1		
Past-year crystal methamphetamine use									
Yes	1.97	1.43, 2.73	< 0.001	1.81	1.17, 2.80	0.008	1.80	1.22, 2.67	0.003
No	1			1			1		
Self-reported HIV status									
Positive	1.56	1.16, 2.09	0.003	1.73	1.19, 2.53	0.005	2.01	1.42, 2.84	< 0.001
Negative/unknown	1			1			1		
Age									
≤ 24 years	1			1			1		
25–34 years	0.77	0.49, 1.21	0.256	0.74	0.43, 1.29	0.293	1.16	0.68, 2.02	0.587
≥ 35 years	.76	0.46, 1.28	0.307	0.93	0.50, 1.73	0.812	1.43	0.77, 2.63	0.252
Education level (highest attained)									
Less than high school	1.53	1.06, 2.19	0.022	1.90	1.24, 2.91	0.003	1.41	0.94, 2.12	0.095
High school or higher	1			1			1		
Employment status									
Not currently working	1.03	0.77, 1.38	0.835	1.04	0.72, 1.52	0.823	0.97	0.68, 1.36	0.845
Currently working	1			1			1		
Individual monthly income (IDR/USD)									
≤ 3.8 million IDR (approx. 285 USD)	1			1			1		
> 3.8 million IDR (approx. 285 USD)	1.23	0.92, 1.65	0.161	1.83	1.25, 2.67	0.002	2.19	1.55, 3.11	< 0.001
Housing status									
Homeless/unstable housing	3.40	1.66, 6.96	0.001	7.44	3.80, 14.56	< 0.001	5.78	2.95, 11.31	< 0.001
Stable housing	1			1			1		
Relationship status									
Married/steady relationship	1			1			1		
Single, never married	0.38	0.24, 0.62	< 0.001	1.00	0.56, 1.79	0.986	0.98	0.57, 1.69	0.952
Divorced/separated	1.05	0.68, 1.63	0.811	2.18	1.33, 3.59	0.002	2.51	1.57, 4.01	< 0.001
Widowed	1.00	0.61, 1.65	0.992	1.00	0.51, 1.96	0.988	2.14	1.25, 3.67	0.005
Dependent children or other dependents									
Yes	1.22	0.91, 1.64	0.180	0.53	0.36, 0.77	0.001	0.63	0.44, 0.88	0.008
No	1			1			1		
Survey city									
Greater Jakarta	1			1			1		
Bandung	0.30	0.20, 0.45	< 0.001	0.74	0.46, 1.19	0.217	0.88	0.60, 1.28	0.506

*OR*, odds ratio; *CI*, confidence intervals; *IDR*, Indonesian Rupiah; *USD*, US dollars; *CESD-R*, Revised Center for Epidemiologic Studies Depression scale

Table 4 presents results from multivariate analyses. In confounder-adjusted logistic regressions, all three syndemic exposures had statistically significant independent effects on the sexual risk outcomes. In particular, IPV (OR = 2.62; 95% CI = 1.80, 3.80; *p* < 0.001), depression (OR = 2.38; 95% CI = 1.67, 3.39; *p* < 0.001), and crystal meth use (OR = 1.66; 95% CI = 1.16; 2.38; *p* = 0.006) each doubled the odds of reporting STI symptoms. Furthermore, higher odds of inconsistent condom use were significantly positively related to IPV (OR = 3.61; 95% CI = 2.03, 6.42; *p* < 0.001), depression (OR = 1.66; 95% CI = 1.00, 2.73; *p* = 0.048), and crystal meth consumption (OR = 1.84; 95% CI = 1.12, 3.02; *p* = 0.016). Lastly, each syndemic exposure remained significantly positively associated with elevated odds of participating in survival sex work, after controlling for sociodemographic co-factors: IPV (OR = 1.85; 95% CI = 1.20, 2.85; *p* = 0.006), depression (OR = 1.83; 95% CI = 1.18, 2.84; *p* = 0.007), and crystal meth use (OR = 1.76; 95% CI = 1.14, 2.70; *p* = 0.011).

**Table 4:** Multivariate associations between syndemic factors, sociodemographic characteristics, and HIV sexual risk behaviors among women who inject drugs in Indonesia

Independent variables	STI symptomatology			Inconsistent condom use			Survival sex work		
	AORs	95% CIs	*p* value	AORs	95% CIs	*p* value	AORs	95% CIs	*p* value
Past-year intimate partner violence									
Yes	2.62	1.80, 3.80	< 0.001	3.61	2.03, 6.42	< 0.001	1.85	1.20, 2.85	0.006
No	1			1			1		
Depressive symptoms									
Yes (CESD-R ≥ 16)	2.38	1.67, 3.39	< 0.001	1.66	1.00, 2.73	0.048	1.83	1.18, 2.84	0.007
No (CESD-R < 16)	1			1			1		
Past-year crystal methamphetamine use									
Yes	1.66	1.16, 2.38	0.006	1.84	1.12, 3.02	0.016	1.76	1.14, 2.70	0.011
No	1			1			1		
Self-reported HIV status									
Positive	1.79	1.28, 2.51	0.001	2.04	1.30, 3.19	0.002	1.98	1.35, 2.91	< 0.001
Negative/unknown	1			1			1		
Education level (highest attained)									
Less than high school	1.38	0.92, 2.07	0.041	2.74	1.68, 4.45	< 0.001	–	–	–
High school or higher	1			1					
Individual monthly income (IDR/USD)									
≤ 3.8 million IDR (approx. 285 USD)	–	–	–	1			1		
> 3.8 million IDR (approx. 285 USD)				2.41	1.56, 3.73	< 0.001	2.59	1.76, 3.80	< 0.001
Housing status									
Homeless/unstable housing	1.90	0.88, 4.11	0.031	4.99	2.44, 10.20	< 0.001	3.47	1.60, 7.54	0.002
Stable housing	1			1			1		
Relationship status									
Married/steady relationship	1			1			1		
Single, never married	0.67	0.39, 1.16	0.156	1.22	0.60, 2.47	0.585	1.07	0.57, 2.00	0.842
Divorced/separated	1.32	0.81, 2.15	0.257	3.00	1.66, 5.43	< 0.001	3.21	1.94, 5.31	< 0.001
Widowed	1.36	0.76, 2.44	0.299	0.93	0.42, 2.08	0.864	2.35	1.24, 4.48	0.009
Dependent children or other dependents									
Yes	–	–	–	0.43	0.27, 0.67	< 0.001	0.49	0.33, 0.72	< 0.001
No				1			1		
Survey city									
Greater Jakarta	1			–	–	–	–	–	–
Bandung	0.36	0.23, 0.57	< 0.001						

Multivariate models for each outcome include the three syndemic variables: past-year IPV, depressive symptoms, and past-year crystal meth use, adjusting for covariates significant at *p* < 0.1 in bivariate analyses with each of the outcomes

*AOR*, adjusted odds ratio; *CI*, confidence intervals; *IDR*, Indonesian Rupiah; *USD*, US dollars; *CESD-R*, Revised Center for Epidemiologic Studies Depression scale

– Not included in the multivariate model

### Additive Effects of Syndemic Exposures on HIV Risk Behavior

Strong additive effects were shown on women’s HIV sexual risk behaviors, after controlling for co-factors (Table 5). The prevalence of STI symptomatology was 11.9% among women who did not experience IPV, depression, or crystal meth use, but increased to 59.7% among women exposed to all three factors. Similarly, with the presence of all three conditions, the prevalence of inconsistent condom use increased sevenfold to 21.6%, from 3.0% among women without these conditions. Moreover, with the concurrent presence of all three exposures, women’s reported prevalence of survival sex work was 24.6%, up fourfold from 6.1% among women without any exposures.

**Table 5:** Predicted probabilities of syndemic exposures on HIV sexual risk behaviors among women who inject drugs in Indonesia

			STI symptomatology		Inconsistent condom use		Survival sex work	
Syndemic exposures			Predicted probability (%)^a^	95% CIs	Predicted probability (%)^b^	95% CIs	Predicted probability (%)^c^	95% CIs
IPV	Crystal meth	Depression						
–	–	–	11.9	6.9, 16.9	3.0	1.0, 5.1	6.1	2.9, 9.2
+	–	–	25.2	17.5, 32.9	9.3	4.8, 13.8	10.3	5.6, 14.9
–	+	–	20.0	13.6, 26.4	5.2	2.3, 8.1	9.9	5.8, 13.9
–	–	+	24.7	17.4, 32.0	4.8	1.9, 7.6	10.2	6.1, 14.3
+	+	–	38.2	30.7, 45.8	15.0	9.6, 20.5	16.0	11.0, 21.0
–	+	+	37.6	29.8, 45.3	8.1	4.4, 11.8	15.9	11.0, 20.8
+	–	+	44.7	37.2, 52.3	13.9	9.0, 18.7	16.5	11.5, 21.5
+	+	+	59.7	54.1, 65.3	21.6	17.4, 25.9	24.6	20.3, 28.9

*CI*, confidence intervals; *STI*, sexually transmitted infection

^a^Adjusted for self-reported HIV status, relationship status, housing status, education level, and survey city

^b^Adjusted for self-reported HIV status, relationship status, housing status, education level, individual monthly income, and having children and/or other dependents

^c^Adjusted for self-reported HIV status, relationship status, housing status, individual monthly income, and having children and/or other dependents

### Interactions

**Fig. 2:**
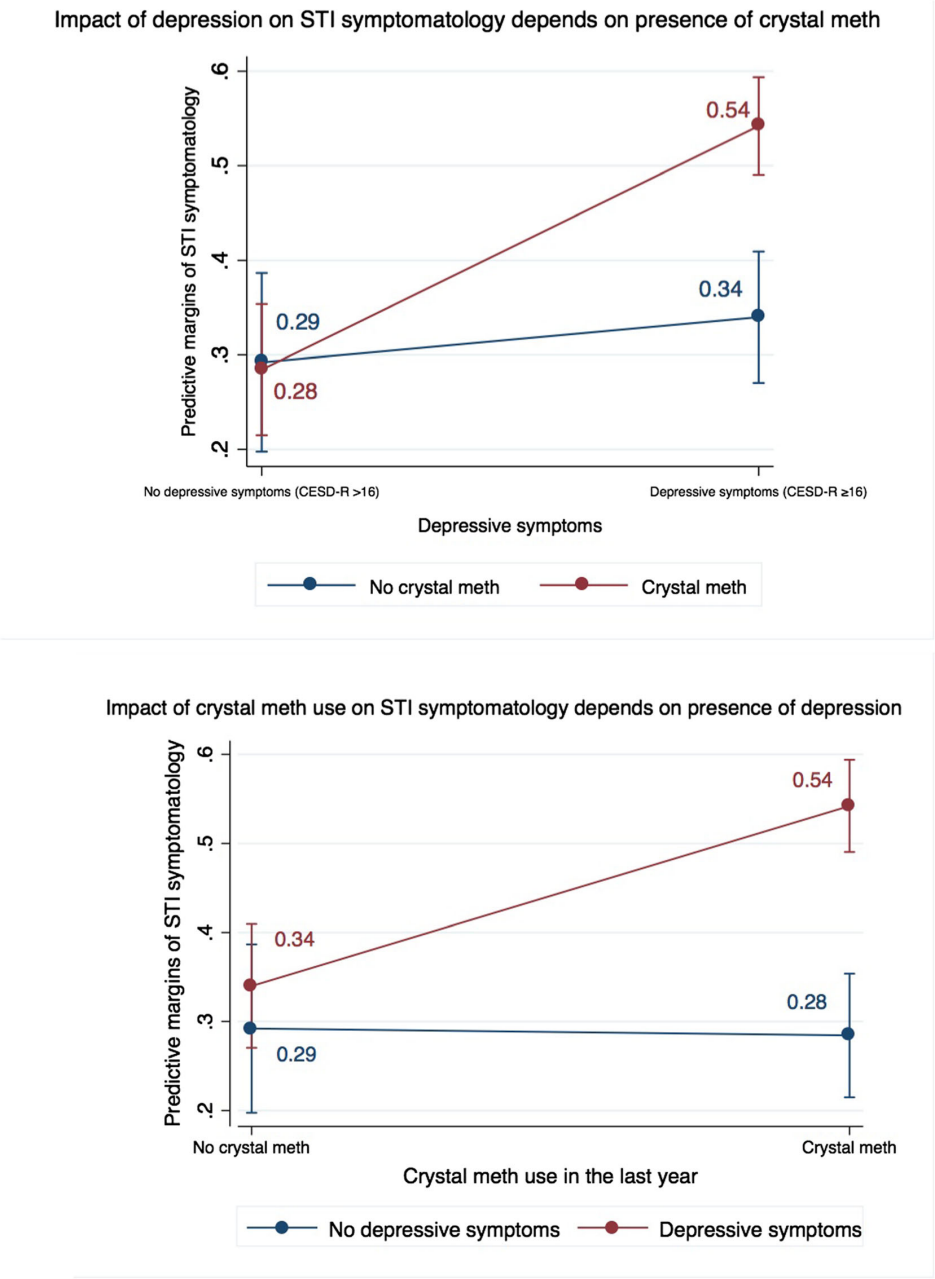
Interacting effects of depression and crystal meth on STI symptomatology among women who inject drugs, controlling for HIV status, education, housing, marital status, and survey city

Interaction analysis using two- and three-way product terms revealed a significant positive interaction on the multiplicative scale between depressive symptoms and crystal meth on STI symptomatology (OR = 2.61; 95% CI = 1.24, 5.48; *p* = 0.011). In other words, the prevalence of STI symptomatology was 54.2% among women who reported crystal meth use and depressive symptoms, relative to 28.4% among women reporting crystal meth only, and 34.0% among women reporting depressive symptoms only. The potential impact of this interaction with and without the presence of depression and crystal meth is visually represented in Fig. 2. No other combinations of syndemic exposures were significantly associated with HIV risk behaviors on the multiplicative scale.

There was also evidence for additive interaction (i.e., synergism). In a series of multivariate logistic regressions, the joint effects of all two-way combinations of syndemic conditions were significantly associated with elevated odds of engaging in HIV risk behaviors, compared to the absence of these conditions (Table 6). Measures of synergism were calculated to better understand the nature of these joint effects. Five out of nine tested joint effects showed evidence of statistically significant greater than additive interaction on at least one of the indices assessed. In particular, a positive interaction was detected between IPV and crystal meth on inconsistent condom use (AP = 0.38, *p* < 0.05). In other words, an estimated 38% of inconsistent condom use among those reporting past-year IPV and crystal meth use was attributable to the interaction between these exposures.

**Table 6:** Main and joint effects of two-way syndemic exposures and additive interactions on HIV sexual risk behaviors among women who inject drugs

		STI symptomatology				Inconsistent condom use				Survival sex work			
		OR (95% CI)^a^	RERI	AP	*S*	OR (95% CI)^b^	RERI	AP	*S*	OR (95% CI)^c^	RERI	AP	*S*
IPV	Crystal meth												
–	–	1				1				1			
+	–	3.47 (1.73, 6.96)***				2.92 (1.03, 8.28)*				2.44 (1.05, 5.65)*			
–	+	2.45 (1.22, 4.92)**				1.37 (0.45, 4.15)				2.17 (0.94, 4.97)			
+	+	6.11 (3.18, 11.72)***	1.19 (− 0.82, 3.20)	0.19 (− 0.12, 0.51)	1.30 (0.79, 2.14)	5.30 (1.98, 14.16)***	2.01 (− 0.33, 4.35)	0.38 (− 0.00, 0.76)*	1.88 (0.72, 4.90)	3.81 (1.75, 8.30)***	0.20 (− 1.57, 1.98)	0.05 (− 0.42, 0.52)	1.08 (0.54, 2.16)
Crystal meth	Depression												
–	–	1				1				1			
+	–	1.01 (0.55, 1.85)				1.79 (0.66, 4.82)				0.99 (0.46, 2.15)			
–	+	1.32 (0.72, 2.42)				1.73 (0.65, 4.64)				1.08 (0.51, 2.32)			
+	+	3.37 (1.95, 5.80)***	2.04 (0.94, 3.13)***	0.61 (0.36, 0.85)***	7.17 (0.26, 198.94)	2.97 (1.19, 7.41)*	0.45 (− 1.07, 1.97)	0.15 (− 0.39, 0.70)	1.29 (0.42, 3.97)	2.28 (1.16, 4.47)*	1.20 (0.32, 2.08)**	0.53 (0.11, 0.94)**	15.96 (0.00, 4.18)
IPV	Depression												
–	–	1				1				1			
+	–	1.98 (1.08, 3.62)*				3.97 (1.42, 11.15)**				1.45 (0.68, 3.07)			
–	+	1.79 (0.96, 3.32)				1.89 (0.63, 5.63)				1.39 (0.66, 2.92)			
+	+	5.78 (3.39, 9.86)***	3.01 (1.11, 4.92)**	0.52 (0.30, 0.74)***	2.70 (1.26, 5.80)**	6.47 (2.51, 16.70)***	1.61 (− 1.25, 4.47)	0.25 (− 0.17, 0.67)	1.42 (0.69, 2.92)	3.04 (1.61, 5.77)***	1.21 (− 0.01, 2.42)*	0.40 (0.01, 0.79)*	2.44 (0.49, 12.24)

*p < 0.05, **p < 0.01, ***p < 0.001. *OR*, odds ratio; *CI*, confidence intervals; *RERI*, relative excess risk due to interaction; *AP*, attributable proportion due to interaction; *S*, synergy index

^a^Adjusted for self-reported HIV status, relationship status, housing status, survey city, and education level

^b^Adjusted for self-reported HIV status, relationship status, housing status, education level, individual monthly income, and having children and/or other dependents

^c^Adjusted for self-reported HIV status, relationship status, housing status, individual monthly income, and having children and/or other dependents

Furthermore, a positive interaction was found between depression and crystal meth on STI symptomatology (RERI = 2.04, *p* < 0.001; AP = 0.61, *p* < 0.001) and survival sex (RERI = 1.20, *p* < 0.01; AP = 0.53, *p* < 0.01). This means that an estimated 61% in STI symptomatology and 53% of survival sex participation among women reporting depression and past-year crystal meth use was attributable to the interaction between these exposures. Particularly, the prevalence of STI symptoms and survival sex work among women who reported both depression and crystal meth use were twice and 1.2 times as high, respectively, as prevalence among women who did not use crystal meth or have depression.

A positive interaction was also observed between IPV and depression on STI symptomatology (RERI = 3.01, *p* < 0.01; AP = 0.52, *p* < 0.001; *S* = 2.70, *p* < 0.01). Namely, the joint effect of IPV and depression was associated with a nearly threefold increase in STI symptoms compared to each exposure’s main effect, with 52% of the presence of STI symptomatology attributed to the interaction of these two exposures.

Lastly, the joint effect of IPV and depression showed a positive interaction on survival sex (RERI = 1.21, *p* < 0.05; AP = 0.40, *p* < 0.05), such that 40% of survival sex work reported by women was attributable to the interaction between IPV and depression.

## Discussion

To the best of our knowledge, this is the first study to quantify the syndemic and synergistic effects of IPV, depression, and substance use on HIV risk outcomes in a community-drawn sample of women who inject drugs in Asia. We found high prevalence of HIV sexual risk behaviors. 58.2–68.8% of women across the two main study cities reported inconsistent condom use, 21.5–52.8% had STI symptoms, and 16.8–18.1% of women engaged in survival sex work. The majority of women reported depressive symptoms, with prevalence ranging from 55.6% in Bandung to 67% in Jakarta, a figure up to 3 times higher than prevalence found among women in a general population sample in Indonesia (22.3%) [43]. Past-year IPV victimization was reported by 55.9–68.9% of women across the two cities. In this sample of women with past-year injecting experience, 50.9–69.1% used non-injection crystal meth. Notably, more than 1 in 4 women experienced concurrent exposure to IPV, depression, and crystal meth use, lending support to the syndemic theory concept of *disease concentration* [26]. The joint effects of most two- and three-way combinations of risk factors were associated with increased odds of engaging in HIV risk behavior. Strikingly, the presence of IPV, crystal meth use, and depression concurrently was associated with a four- to sevenfold elevation in women’s reported prevalence of sexual risk behaviors.

Our findings suggest that the overlap and interaction of these key syndemic factors has a significant impact on HIV risk behaviors in this sample of Indonesian women who inject drugs. Specifically, experiencing depressive symptoms in conjunction with using crystal meth nearly tripled women’s odds of reporting STI symptoms. Moreover, most two-way effects of syndemic factors showed a greater than additive interaction. Notably, 38% of inconsistent condom use among women who experienced past-year IPV and used crystal meth was attributable to the intersection of these risk factors. Furthermore, the presence of STI symptomology among women exposed to both IPV and depression was more than three times higher than the prevalence of STI symptoms among women without either exposure. Likewise, the interaction between crystal meth use and depression accounted for 61% of reported STI symptomatology and 53% of survival sex work among women with both conditions. These sets of interactions reflect multiple mutually reinforcing risk pathways. Women who use drugs, particularly those in intimate partnerships with drug-using partners, may engage in survival sex work for a variety of reasons, including the need to provide materially for their families, support their and/or their partner’s drug use, and cope with the traumatic and economic consequences of being in an abusive relationship [73–75]. Unsafe sex work environments, where coercive sex is common and substance use abounds, may further exacerbate women’s vulnerability to HIV transmission [76]. For instance, in a recent study in Indonesia, women reported frequently using crystal meth use to cope with transactional sex with multiple sexual partners, and experiencing vaginal injury as a result of aggressive and prolonged intercourse [38]. Increased use of crystal meth may restrict women’s capacity to negotiate risky situations and cloud their ability to recognize an escalating situation [46]. The psycho-pharmacological effects of stimulants can also trigger IPV perpetration by intensifying feelings of paranoia, jealousy, and irritability, as well as impair judgment—effects that in turn increase the likelihood of IPV and decrease the ability to use condoms [77]. Meanwhile, the trauma associated with experiencing IPV may lead to depression, since traumatic events can trigger fear, stress, and isolation, which can subsequently cause depressive symptoms [14]. While previous studies have documented the concentration of these health and psychosocial problems among women who use and inject drugs [8], this study extends the field by demonstrating that a substantial proportion of variation in women’s HIV risk behaviors may be explained by interactions among syndemic IPV, crystal meth use, and mental health challenges.

Strengths of this study include its large sample size and the community-based nature of the sample. A notable limitation of the present study is its cross-sectional design, which measured HIV risk outcomes and independent variables retrospectively and at one data point. Thus, while this study provides indications of areas where future longitudinal and intervention research could helpfully be focused, its study design characteristics limit the ability to determine temporality. Future research with longitudinal designs is urgently needed in Asia to build on the present study and further elucidate temporal relationships between syndemic factors and HIV risk. A second limitation of the study is its reliance on self-reported measures, which are prone to recall and social desirability bias, particularly in relation to illicit or illegal behaviors (i.e., drug use) and taboo topics (i.e., sexual practices, IPV, and HIV) [78]. To reduce potential biases associated with self-report, this study utilized trained female peer interviewers, a proven strategy for improving rapport and trust between interviewers and participants, thus increasing the chances that participants respond honestly [79, 80]. Furthermore, the research team sought to decrease participants’ discomfort and improve confidentiality by ensuring that interview locations were, in every case, private, safe, and chosen by the participants themselves. Thirdly, it is important to acknowledge that measures of additive interaction calculated using ORs, as has been done in this paper, only approximate relative risk ratios (RRs) in cohort studies. In their analysis of the validity of RERI, AP, and *S* as measures of interaction when ORs are used as approximations for relational risks, Kalilani and Atashili (2006) observed that ORs may produce unreliable estimates depending on the measure of interaction used, the type of interaction present, and the baseline risk of the outcome [29]. Specifically, for all three metrics, the interaction assessed was consistently of higher absolute magnitude when measures used ORs, compared to measures derived from RRs, especially when the study outcome was not rare. The RERI metric, although most widely reported in analyses of additive interaction in epidemiologic studies, appears to be the most unstable. When comparing greater than additive interaction measures derived from ORs with those calculated from RRs, the increase in magnitude of interaction with each increase in the baseline risk was most pronounced for the RERI and *S*, and least pronounced for the AP metric [29]. Furthermore, it has been shown that in analyses of additive interaction that adjust for confounding in regression models, as is the case in this paper, *S* is the most robust among the three indices as it does not vary across strata of the additional covariates [81]. Given that the outcomes in the present paper are not rare and all analyses adjust for confounding, caution is thus advised about affirming the presence of greater than additive interaction based on exclusively on the RERI metric. To minimize this limitation, we followed Kalilani and Atashili’s recommendation to assess and report all three indices in order to provide a range in the estimation of additive interaction and allow for a careful consideration of any inconsistencies among them [29]. No discrepancies were observed between the three indices in terms of the type or direction of interaction for any of the nine analyses of additive interaction assessed in this paper. Nevertheless, in the absence of longitudinal data confirming the risk factor relationships observed here, the findings should be interpreted with caution. An additional constraint is the inability to test for additive interactions beyond two exposures due to limitations inherent in tests of synergism [82]. Furthermore, RDS analysis methods and accompanying software were designed for point estimation but do not lend themselves to multivariate analysis [64]. Thus, while the city-level point estimates fulfill RDS theoretical assumptions, the unweighted multivariate findings cannot be generalized beyond the population under study. Other limitations include the reliance on self-reported measures, which may be subject to recall and reporting bias, particularly as related to illicit, illegal, and intimate behaviors.

Notwithstanding these caveats, the data presented here contribute to the empirical evidence for risk factor interaction among women who inject drugs. Our findings show that, in a sample of drug-using women in Indonesia, IPV, depression, and crystal meth use co-occur and interact synergistically to amplify the burden of HIV risk. These findings have important public health implications. Considered in tandem with previous research showing that the syndemic factors of IPV, depression, and substance use are also mutually causal [8], this study contributes to the problem theory for drug-using women’s HIV risk behavior in a middle-income setting. The selection of these particular syndemic factors was informed by previous research pointing to the SAVA syndemic as the key locus of overlapping risk for this population in other settings, the dearth of quantitative research on this topic in Indonesia, and the range of variables available in our dataset. Syndemic theory, and, specifically, its concept of disease interaction, involves multiple levels of analysis, as pointed out by several epidemiologists [24–26]. Understanding the adverse impacts of structural conditions that could be mitigated through longer-term cultural, economic, or legislative interventions at the level of populations using multi-level modeling could have greater impact than modifying syndemic factors at the behavioral, interpersonal, or network levels alone. Large-scale social forces operating at the policy and structural levels, such as drug legislation, poverty, mass incarceration, and beliefs about gender roles, may play a key role in producing, perpetuating, and potentially ameliorating negative health outcomes [26]. For instance, power imbalances within relationships and men’s beliefs about societal gender roles are established risk factors for IPV in several settings [83, 84], and are likely to impact violence against women, and in turn HIV risk, in contexts like Indonesia, where women are structurally disadvantaged and IPV tends to be socially tolerated. Tsai and his colleagues have argued that if such harmful structural conditions were diminished through cultural, economic, and legislative changes, individual and relational risk factors may also be efficiently mitigated at the population level [25]. To this end, future research modeling the impact of locally-relevant structural factors on the syndemic associations observed here would be of great utility in mitigating women’s overlapping vulnerabilities.

In the Indonesian context, the implementation of responsive and tailored interventions for women who use and inject drugs is hindered by structural and legal barriers [86]. The criminalization of people who use drugs tends to drive the most marginalized members of the population further away from health and support services due to fear of arrest [87]. High rates of gendered discrimination and abuse faced by women who use drugs in both health and criminal justice settings may further contribute to their adverse health outcomes [40]. Thus, in order for harm reduction, mental health, HIV prevention, and IPV interventions to be accessible and beneficial to this highly stigmatized community, they must be supported by conducive policies. Such policies should be aligned with improving the health and psychosocial well-being for vulnerable drug-using populations. Future research could inform this policy goal by using modeling to approximate the ways in which conducive drug policies could reduce the clustering of comorbidities among marginalized women in low- and middle-income contexts.

In the absence of tailored multicomponent interventions that target the full scope of syndemic vulnerabilities, improving women’s health and psychosocial well-being may require, in the first instance, improved access to low-threshold health care, with adequate referral links to mental health, reproductive health and rights, and IPV support services. A sensible first step in this direction could be adjusting existing harm reduction and other HIV prevention programs to enable greater uptake by drug-using women. This could be achieved by hiring more female staff, especially female peers, providing on-site childcare support, and expanding fixed-site health care to include mobile services, among other low-cost modifications [85].

In conclusion, by contributing to the empirical basis for the syndemic interactions between mental health, IPV, and substance use among women who inject drugs, this study highlights the urgent need to optimize existing health systems to better address the needs of key populations. Implementing effective interventions to address this challenge requires not only programmatic improvements, but attention to the broader social and policy contexts that can reduce adverse health outcomes.
